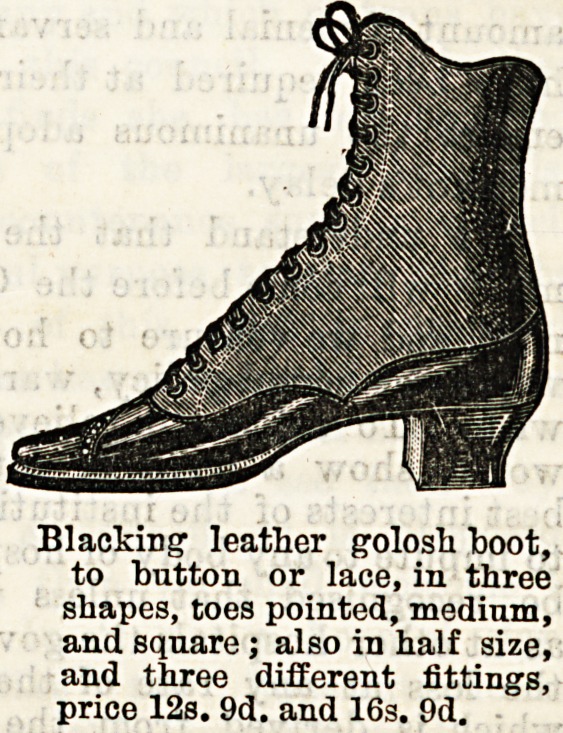# The Hospital Nursing Supplement

**Published:** 1894-10-27

**Authors:** 


"The Hospital" Nursing Mirror.

Being the Extra Nursing Supplement of "The Hospital" Newspaper.

[Contributions for this Supplement should be addressed to the Editor, The Hospital, 428, Strand, London, W.C., and should have the word "Nursing" plainly written in left-hand top corner of the envelope.]

### News from the Nursing World.

### ST. THOMAS' TRAINING SCHOOL.

Of the thirty-three nurses trained in the Nightingale School last year thirty-two have been transferred to the hospital nursing staff, and one probationer has gone to learn district nursing as a preparation for becoming a Queen's Nurse. Lectures have been given to the Nightingale probationers by Dr. Ord, Dr. Sharkey, and Mr. Lunn. At St. Marylebone Workhouse Infirmary eleven nurses have completed their training during the year, and all remain on the staff of that institution.

### THE LONDON HOSPITAL.

The lectures given annually to the probationers at the London Hospital are now in progress. The Matron's course on Nursing began August 29th, and, will be succeeded by the lectures of Mr. Mansell Moullin on Elementary Anatomy. The sick room cookery classes and demonstrations have been found to work exceedingly well at the London, and Miss Lückes is to be congratulated on the inauguration of this valuable branch of instruction. Examinations connected with it will take place in November, and doubtless the results will be as satisfactory as those which have followed the previous courses. The *London Hospital Gazette* has issued an excellent third number, and the editors have certainly cause to be proud of a new journal which is cordially welcomed by all old Londoners. Its literary merit is considerable, and it is excellently got up. A charming etching by Mr. Hutchinson, jun., illustrates an interesting article penned by himself.

### DISTRICT NURSING IN SOUTH LONDON.

The funds of the South London District Nursing Association appear to need supplementing by increased subscriptions. Canon Erskine Clarke (Chairman) and Mr. J. F. Schwann (Hon. Treasurer) are appealing also for blankets, sheets, shirts, flannel jackets, water and air pillows, old linen, and invalid appliances for the sick poor whom the district nurses attend. The home is situated in Marmion Road, Lavender Hill, S.W., and the nurses work entirely under the local medical men.

### POSTPONED PAYMENTS.

A point of some interest was raised at a recent meeting of the Uxbridge Joint Hospital Board with regard to the payment of the nurses. Although the Board, it is said, allows a sum, in advance, for this purpose, the nurses' claims have not been settled promptly. In one case, as reported by the local press, six weeks elapsed before payment was made, and in another a nurse had been seriously inconvenienced by the delay. The Superintendent said that he had been asked in more than one case not to pay the nurses, but to send the money to the institutions from which they came. This hardly excuses the delay in payment, and although there may be a good reason for it, it is obvious that the practice might cause inconvenience. It would be interesting to know the custom at other institutions.

### MISS NIGHTINGALE AND QUEEN'S NURSES.

The establishment at Aldershot, Plymouth, and in Ireland of trained nurses for the families of soldiers and sailors has met with the cordial approval of Miss Nightingale. Addressing Colonel Gildea on the subject, she particularly commends the supervision of the Inspector of the Queen Victoria Jubilee Institute as "a valuable safeguard" in connection with these nurses. It is with great regret that our readers will learn that Miss Nightingale's explanation of her delay in writing earlier on this subject was due not only to press of work but to ill-health.

### CHARITY AT BRADFORD.

Hitherto the gratuitous nursing of the sick poor in their own homes has been partially maintained by the earnings of the private nurses of the Bradford Institute; the district nursing having become a most popular branch of the work, although adequate independent pecuniary support has not been forthcoming. Dr. Goyder remarked at an "At Home" recently held at the Institution, that the application of the whole profits of the private nurses' earning to the work of charitable nursing "was neither just nor politic." During the last few months the salaries of the nurses who attend paying cases have been increased, and their comfort has also been augumented by additions to the Nurses' Home. The expenses of the building appear indeed to have been defrayed by the nurses' earnings, which are now exhausted. The interest of ladies residing in the neighbourhood has been claimed in the present crisis, and it is desirable that steps should be taken to have the district work organised as a separate "charity," not as one carried on at the cost of working nurses. The latter are certainly entitled to receive and dispose of their own earnings in their own way. The entertainment at the Home on the 12th inst. was well attended, the Mayor and many other friends of Bradford charities being present on the occasion. No doubt a reasonable and just scheme will shortly be organised by the liberal inhabitants whose co-operation was ably requested by the speakers at the "At Home."

### MEDICAL WOMEN IN EDINBURGH.

The Edinburgh University Court having resolved to admit women to graduation in medicine and surgery, have, in accordance with Ordinance No. 18 (Section III., 4), recognised the Edinburgh School of Medicine for Women, Surgeon Square, and the Medical College for Women, 30, Chambers Street, Edinburgh, as medical schools whose courses of instruction qualify women for graduation in medicine. Further, students from other Universities are admitted for graduation, in accordance with certain rules and regulations. We congratulate the Medical College for Women in Edinburgh on the success which has attended its efforts to secure these privileges for lady students. Would-be candidates cannot do better then enrol themselves under the banner of a school which promises to hold a first place amongst the centres open for female medical education. All particulars can be procured by writing to the Secretary at the Medical College for Women, 30, Chambers Street, Edinburgh.

### SKILLED NURSING IN ABERDEEN.

Dr. Angus Fraser presided at the quarterly meeting of the Aberdeen District Nursing Association recently held at the Nurses' Home in King Street. There was a large attendance, much interest being exhibited in the Association, of which Miss Peter, Inspector of the Queen's Jubilee Institute, reported that "The nursing is excellent." Special nourishment and a night nurse are supplied when needed by a fund raised by the Aberdeen District Nursing Dorcas Society, the Aberdeenshire Needlework Guild, and the Aberdeen Ladies' Sanitary Association, and the work of Miss Armstrong amongst the sick poor in their own homes is cordially appreciated by her patients.

### FEVER NURSES IN IRELAND.

Fever nursing does not seem to be considered an attractive calling in Ireland, for there were only two applications made for the vacant post of assistant nurse at Ballymonery Fever Hospital. One of the women failed to appear on the appointed day, and the other, a girl of fifteen, a recently-discharged patient was considered too young and inexperienced for such responsible work; therefore no appointment was made.

### COPENHAGEN.

The news of the death of Frou Emmy Lange was received with much regret by all classes in Copenhagen. She was the fourth woman doctor in Denmark, which only possesses seven. She became a student in 1880, and eventually married Dr. Lange. Her career was a successful one, and having passed her final examination she afterwards worked both at the Alminsdeliz and at the Royal Frederik's Hospital. She was equally liked and respected by professors, fellow-students, and patients, for she was both charming as a woman and clever as a lady doctor.

### MIDWIVES IN SWEDEN.

The number of midwives in Sweden amounted at the beginning of last year to 2,553, and during 1893, 107 pupils have studied and, after the usual examination have been duly certificated. Of these, 63 passed at the Stockholm institution, 34 at Gothenburg, and 10 at Lund, 91 evincing sufficient knowledge to become certificated to use instruments at confinements.

### PRIVATE NURSES IN SYDNEY.

New South Wales is not free from the women who, with some little hospital experience, have the effrontery to pose as trained nurses. A serious complaint comes from the colonies to the effect that the former are injuring the position of the latter. The adoption of uniform, and the asserted possession of "hospital experience," are accepted by the unsuspecting public as credentials of competency! This is on a par with the Englishwomen who, on the strength of three or six months' midwifery or "monthly" teaching, set up in rural districts under the misleading designation of parish or village "nurse."

### MATRIMONIAL CONTINGENCIES.

Dr. William Osler, in addressing the Alumni Association of the Harvard Medical School, referred to the custom at the Johns Hopkins University of male and female students working in the same school, and surprised his hearers by stating that at the end of one short session 33·3 per cent. of the ladies were going to be married. Dr. Osler, apparently, considers this fact to indicate that co-education is not the success which he had previously expected to find it.

### PITTSBURG.

The manager of the West Penn Training School, Pittsburg, Pa., determined this summer to receive male probationers desirous of instruction in sick-nursing. All the young men who have yet entered propose eventually to take up medicine. Miss Milne, the new superintendent of nurses, has given the subject much attention, and the scheme should succeed. Unfortunately the first male pupil in the school has fallen a victim to typhoid, and his death on August 19th cast a gloom over the institution in which he had already gained much affection and respect.

### INFANTS IN PARIS.

A new *crèche* is about to be organised in Paris in one of the poorest and most densely crowded quarters of the city. It is said to be urgently required, and has therefore been authorised by the Council of the Seine. Children from eight days to three years of age will be eligible for admission.

### SHORT ITEMS.

The nursing arrangements at Hampstead appear very unsettled, according to the local press. The chairman frankly owned at a recent meeting that the board had never got on so well since they left the association which had for so many years supplied them with nurses.—At the Newbury harvest thanksgiving £23 3s. 10d. was collected at St. Nicholas' Church for the Parish Nurse Fund.—The Dudley Board of Guardians has been officially recommended to appoint two additional nurses and a night attendant for the imbeciles in the workhouse.—The Sorn and Catrine Nurse Association has issued its report, the work of Nurse Heath being considered altogether satisfactory. —There seems a chance that the recent acquisition of more land will enable the Shoreditch Guardians to provide improved accommodation for their nurses.—A motion to retain a pauper as ward mistress over the female lunatics at Lorne Workhouse, at a salary of half-a-crown a week, was lost by one vote, no appointment being made.—The Bristol Board of Guardians has decided to take probationers for two years' training.—A new nurses' home has just been completed at Middlesbrough for the Nursing Association. It was formally opened by Mrs. Dorman.—Mr. T. Gold Edwards has been elected to succeed the late Dr. Turnour as President of the Denbigh Jubilee Nursing Institute.—In the reports on the health of the Ameer attention is called to the presence in Cabul of Dr. Lillias Hamilton, who has been installed in the Royal harem.—A gold pencil case was presented to Dr. Neilson, of Nottingham, by the ladies of his ambulance class.—Subscriptions are being raised for the purpose of securing a trained nurse for Appleby. —The District Nursing Branch of the Croydon Nurses' Institute requires more liberal support to enable the work to be carried on efficiently.

### Notes for Nurses on Antiseptic Surgery.

By William Horrocks, M.B., F.R.C.S., Hon. Surgeon to Bradford Infirmary.

### III.—ORDINARY ANTISEPTIC DRESSING.

The objections to the early method of applying antiseptic treatment were soon apparent to Lister. It was obvious, that the guard of lint, if held over the wound, made it difficult to see the field of operation—if held over part of the wound, it did not prevent the access of the ever-present germ. The constant irrigation with carbolic lotion made operation difficult, as the lotion stained with blood obscured the parts. In addition the constant soaking of tissues for long periods caused considerable irritation of the part, and in some cases general carbolic acid poisoning. Another great objection to this method was the lead plate, which was difficult to apply to parts, and still more difficult to prevent the volatile carbolic acid from escaping from under its cover.

### Abandonment of Early Method.

Lister soon gave up this method, and introduced his method in its better-known form. The early trial is interesting from more points than one.

In describing the earliest method of antiseptic dressing the wounds were spoken of as being drained and the vessels tied with catgut ligatures. It would be well to consider these two subjects.

### Introduction of Drainage Tubes.

Drainage of wounds was not invented by Lister. It was due to a French surgeon, Chassaignac, who from observing the tiles used to drain marshy land, applied the same means to remove the excess of fluid from wounds or abscesses. Drainage was an absolute necessity in the earlier Listerian treatment. The irritation of the carbolic lotion caused an abundant exudation of watery fluid (serum) during the first twelve hours following the operation. If this was not allowed to escape, it accumulated and separated the wound surfaces, hindering the process of healing.

Lister introduced drainage tubes of red rubber, which is free from sulphur; the tubes being of various sizes, and perforated at intervals by openings one-third of the calibre of the tube.

### Their Uses.

The inner end of the tube dips into the deepest part of the wound, and the outer end, which is if possible dependent, is cut flush with the skin. Lister recommends that several small tubes should be used rather than one large tube. The tubes are left in until the third day, when they have formed a passage into which they easily go, they are then shortened or returned unaltered, after thorough cleansing with carbolic lotion. The outer end of the tube is secured by silk or a safety pin. This is especially necessary in draining large cavities, as the tube may slip in and be lost.

### Catgut Drains.

Mr. Chiene, of Edinburgh, introduced another form of drain, utilising the force of capillary attraction. You all know that if the end of a fine skein of wool is dipped in water, the water rises between the threads, rising by what is known as capillary attraction. The same thing is illustrated by the water in a glass, which is higher on the surface where it touches the glass than in its centre. Mr. Chiene used a number of threads of catgut, which he bound together and placed their ends in the deeper part of the wound, while he brought their free ends to the surface. He thought that this would allow capillary drainage during the first twenty-four hours, and afterwards the threads would become absorbed. With the harder forms of chromic gut the strands do not swell, but allow drainage between the fibres. The ordinary carbolised catgut swells up during the first twenty-four hours, and acts as a plug rather than a drain. A much better capillary drain is a strand of horsehair which has been thoroughly cleansed and carbolised. The hairs do not swell in water, and may be withdrawn hair by hair as they cease to be required.

### Absorbable Drainage Tubes.

Another form of absorbable drainage tube was introduced by Dr. Neuber, of Kiel, and improved by Dr. McEwan, of Glasgow. The thigh bone of the chicken is softened by immersing it in hydrochloric acid, the ends are cut off and the central narrow cavity is hollowed out. Holes are now cut out at intervals and the tubes kept in 1 in 20 carbolic lotion. These tubes are absorbed by the tissues as the wound heals. They are useful for eight days.

In tying vessels Lister introduced a great advance. Before his time the arteries were tied with waxed dentist's twist, and the ends were left hanging from the wound to allow the ligature to be taken away, when separated from the vessel. I have only seen one wound in which the vessels were tied in this way. It was a sailor, whose thigh was amputated by a Portuguese surgeon in Sierra Leone. The ends of the ligatures were hanging out during his voyage from Africa, and only came away about six weeks after the amputation. It is evident that a ligature which has to separate in this way is very undesirable.

### Sir Astley Cooper's Ligatures.

Sir Astley Cooper was the first, who attempted to use a ligature which would be absorbed, when it ceased to be needed to occlude the vessel. He used catgut or fiddle string but without any previous preparation, and left the end of the ligature hanging from the wound. The unprepared catgut, when left in the wound, soon became soft, and not being made antiseptic in any way, infected the wound.

Sir Astley Cooper gave up the use of the catgut ligature; it was re-introduced by Lister, but with this difference the gut was rendered antiseptic. To prepare the gut for ligatures Lister dissolves 1 part of chromic acid, and 200 parts of pure carbolic acid in 4,000 parts of water; selected catgut, *i.e.*, strips of the intestine of a cat, are placed in this for 48 hours, they are then removed, dried, and stored in bowls contatning 1 in 5 carbolic oil. In some cases the dry chromic gut is used. This is immersed in lotion for half an hour before the operation. Silk is also used. It is prepared by boiling for half an hour in 1 in 40 carbolic lotion, and storing in carbolic lotion 1 in 40.

### A New Society.

In Copenhagen a society has been working for some little time, having for its object and name "The Nourishment of Infants." The results so far appear very satisfactory. It supplies "children's milk"—from thoroughly controlled cows of the "Copenhagen Milk Supply," probably one of the best organised dairy companies in the world—in a thoroughly sterilised condition. Each bottle contains the average meal of an infant, and the milk is delivered in four different degrees of dilution, according to the customer's requirements. The milk is to be obtained from apothecaries and dairies, the bottles being sold in a kind of cruet stand. The empty bottles are returned to the same places without being cleaned, and the only trouble for the mothers or nurses is to warm the milk a little. The cost and the labour entailed is considerable; the cleaning of bottles, weighing and mixing of milk, and especially the sterilising by boiling. The society, however, brings the boon of milk, free from germs, within the reach of poorer people, although it does so at a sacrifice. A set of eight bottles, suitable for children during the first six months, is delivered at the price of 15 öre or 2d. per day, the society adding about 3d. per day to this payment. In order to obtain the milk at this reduced price the doctor of the family has to fill up a printed form answering various questions.

### Male Nurses.

TRAINING IN THE MEDICAL STAFF CORPS.

A man wishing to join the Medical Staff Corps must be between the age of 18 and 25, of good character, able to read and write, and understand written instructions. When a man enlists he is sent straight to the depôt at Aldershot, and usually remains there from three to six months, during which time he goes through about two months' squad and company drill, one month's musketry and firing, and has to attend forty-five lectures of one hour each; also one hcur is spent each day in ambulance drill, one hour in exercise and examination on the lecture of the previous day and bandaging. The subjects of the lectures are anatomy, physiology, treatment of the sick, application of dressings, management of wards, and the names and uses of surgical instruments. When a man is in the depôt as a recruit his pay is 1s. 2d. per day, out of which 3½d. is stopped for groceries and 1d. for washing daily, also 1d. per month for hair cutting, which leaves his total clearings at 9½d. a-day during his recruit's course. After he has passed his course of instruction satisfactorily he is rated a third class orderly, and receives 4d. a day more pay. All through his service a man is provided with uniform, boots, and ward shoes, and a careful man generally finds he can make the two former do, but most of them need to buy extra shoes, which are brown canvas. Two shirts, two pairs of socks, two towels, brushes, razor, gloves, &c., are supplied to a man when joining, but he has to renew them whenever needed at his own expense. After leaving Aldershot he goes to a station hospital, where by good conduct, cleanliness, and efficiency he can be further advanced to second class or first class orderly, by which advancement he gains an additional 2d. per day for each grade, so that by the time a man has reached his full grade he is receiving after having been stopped 3d. per day for groceries and 1d. for washing, 1s. 6d. a day clear; this is paid weekly. After a man has gone two years without a regimental entry on his defaulter-sheet he becomes entitled to a good conduct badge, and after six years to a second, in twelve years to a third, sixteen years to a fourth, and twenty-one years to a fifth. He gets 1d. a day extra pay for each badge up to the fifth. A man who neglects his duty or is disobedient or untidy, and unpunctual, or does anything else wrong, is not discharged as a nurse in a civil hospital would be, but is liable to punishment under military law; it may be so many days' confinement to barracks, or for more flagrant offences, even cells. When a man is undergoing punishment he is stopped his departmental pay from the day he is made a prisoner to the last day of his punishment. The rates of stoppage of pay are: 3rd class orderly, 4d. per day; 2nd class orderly, 6d. per day; 1st class orderly, 8d. per day. A man enlists for three years with the colours and nine years on the reserve. At the end of his three years' service he receives £1, and can take any work he pleases during the nine years. During that time he receives pay at the rate of 6d. a day, and is liable to be recalled to nursing duties in case of war. Or a man may enlist for seven years with the colours and five years on the reserve. At the end of his seven years' service he receives £21, and the five years' reserve under the same conditions as the nine years. If a man prefers remaining in the Army he can re-engage at seven and twelve years. An orderly at a station hospital rises at six or half-past six a.m., has to be in his ward at seven a.m., brings up the bread and butter for the day, also the breakfast-tea which is made in the cook-house; he has to sweep and dust his ward, make the beds, prepare for the medical officer's visit, and go round with him when the visit is over; he has to take the prescription-book and empty bottles to the surgery, make out and give in his list of diets for the day, draw extras (such as eggs, lemons, &c., and stimulants), then on about two days a week he will probably have one hour's drill, and each day a short class of instruction from the medical officer; he also has to see to new admissions, and the getting-up of dinners; he has one hour for his own dinner and a short parade, returns to his ward and remains on duty till five p.m., when the night duty men relieve him.

### Matrons in Council.

It would appear from the semi-official statements which have been made public concerning Miss Isla Stewart's proposal to form yet another association, to be entitled "Matrons in Council," that the opposition tbe proposal has created has not been without effect. If there is to be a "Matrons' Council" at all, it should be representative of all that is best and highest and most influential in England to-day. As the matter stands at present, when judged by this test, the Matrons' Council, so far as the leading English matrons are concerned, occupies the position of Miss Isla Stewart *contra mundi*. Miss Isla Stewart is matron of one of our largest hospitals and it is incumbent upon her to realise the responsibility which attaches to that position, by stating plainly, at the meeting on November 1st at St. Bartholomew's Hospital at three p.m., how many and which matrons have applied to become members of this council. It is further incumbent upon her if she finds she has not received the support of the matrons of the largest hospitals, without whose support and countenance such a council must be ridiculous for any useful purpose, to state that she realises the force and truth of this, and is prepared not to proceed further until she has succeeded in enlisting the aid and authority of the majority of her sister matrons of eminence. If the representative matrons see their way, if the matrons believe such a council as is proposed, to be a useful establishment, then we shall welcome it —welcome and support it. It is not a hopeful sign, however, to find that, unlike the Superintendents' Association in the United States, this council is to include women who are or have been matrons of hospitals, or superintendents of institutions; in other words, membership is not to be confined in the first instance, as it is in the United States, to the matrons attached to the principal training schools, or even to matrons of other reputable hospitals and institutions, but is to be open to all and sundry who can claim to have at any time or, apparently, anywhere held a post in an institution to which the name of matron or superintendent was attached. We hope the draft bye-laws which contain these powers will not be adopted before they have been materially modified. The discussion which is promised upon the proposal to admit sisters of wards to the Matrons' Council will be awaited with interest. Whatever the result, we hope that no premature and hasty action will be taken, as a contrary course must bring discredit upon the new association, and deprive it of all authority and usefulness.

### Presentation.

On leaving the Northern Infirmary, Inverness, for her new appointment at Bona, Miss Keir received a pretty time-piece from the nursing staff, and a pair of pictures from Miss Macconnachie, the Matron.

On the departure of Mrs. Warren, assistant matron of the Chelsea infirmary, she was presented by the staff with a handsome travelling clock, a massive silver clasp, and an afternoon tea service. Mrs. Warren carries with her to her new post at Caterham the esteem and affection of all with whom she has been associated.

Miss M. M. Macfarlane, who was recently appointed Matron of the Victoria Infirmary, Glasgow, has been presented with a silver teapot, silver salver, and umbrella by the med ical and nursing staff of Barnhill Hospital as a token of their respect and esteem. Dr. Core, in making the presentation, referred to the cordial relationship which had always existed between the members of the staff and Miss Macfarlane.

### Addenbrooke's Hospital, Cambridge.

### WARDMAIDS WANTED.

Last week we expressed satisfaction that the subject of wardmaids had been brought before the weekly board of this hospital, and we ventured to express a hope that they would be introduced without further delay. How urgent is the demand for this reform at Addenbrooke's Hospital will be realised when it is stated that, although the hospital has received an income of £11,452 from the probationers' payments since this system was introduced in 1878, only £4,724 has been paid for skilled help during the same period; in other words, a profit of nearly £7,000 has been made by the hospital out of the probationers, all of whom have paid for their training. Such a profit could never have been realized had not the probationers been compelled to do an amount of menial and servants' work which ought never to have been required at their hands. This fact alone should ensure the unanimous adoption of wardmaids without a moment's delay.

We understand that the question of employing wardmaids will come before the Court of Governors on Monday next, and we venture to hope that as an act of justice, as well as of sound policy, wardmaids will be introduced forthwith. To refuse to relieve the probationers to this extent would show a want of intelligence and attention to the best interests of the institution, which it would be an insult to impute to any body of hospital governors. It must further be recognised that unless wardmaids are now employed as at other hospitals the governors must be prepared to face the loss at any rate of the greater portion of the income which is derived from the payments made by the probationer nurses.

If there be any narrow economists amongst the governors of Addenbrooke's Hospital, as there are at most hospitals, who often, though wrongly, imagine that probationers are a great expense to a hospital, we would ask these gentlemen to consider the sum by which they will have to increase the expenditure of the hospital, if it is found, owing to the absence of wardmaids, that the probationers have to be paid for their work, as they most certainly will have to be paid, in the near future should they not be relieved from the menial and servant's work which they are at present compelled to do, to the injury of the reputation and credit of Addenbrooke's Hospital which deservedly holds a high place in other respects amongst the great hospitals of this country.

### Everybody's Opinion.

[Correspondence on all subjects is invited, but we cannot in any way be responsible for the opinions expressed by our correspondents. No communications can be entertained if the name and address of the correspondent is not given, or unless one side of the paper only be written on.]

### DISTRICT NURSING.

"A Reader" writes: We are just setting up a district nurse in this parish, and should be very glad to receive from anyone who is at the head of a similar work in a country district the rules and regulations which are there in force, or any other guidance in the matter.

Miss Alice K. Coburn writes: With reference to the article on District Nursing in The Hospital of October 6th, inconsistency shows itself in more ways than one, and I should like to know if it is consistent of the Rural Branch of the Q.V.J.I. with their high standard to inspect and receive monthly reports from women whose "knowledge is only perfunctory"?

### NURSING IN PARIS.

"Another Levick Nurse" writes: Having read the last two letters in The Hospital regarding the treatment of nurses at the Levick Institution, I feel I should like to add my experience of the same. I myself worked for them for twelve months and received six months' salary, the agreement being thirty pounds a year and my passage to and from England. I was forced, like my fellow-nurses, by the treatment, viz., starvation, and no water to drink, to apply to the British Consul to assist me to England, which he very kindly did. It having come to my knowledge that a report is in circulation that it was entirely our own fault that we left Paris without being paid, I am in a position to state that this is not correct. We tried by all means to get our salaries, and the reply was that Mr. Levick had no money. My salary (£15) and my passage to and from England, is still owing me. The conduct of the Matron to friendless girls was disgraceful. What were we to do? They offered us sleeping accommodation and no food, and we were without means.

### "OVER THE HOSPITAL WALL."

We print the following exactly as we received it: In answer to last weeks Over the Hospital wall, the Women calling herself Catherine Bassett has this to say she was not engaged by the Matron. The Committee found her Testimonials satisfactory and engaged her for the post. The Matron is very curious to know every ones secret and she found out mine by reading one of my letters which came accidentaly into her hands she took the opportunity to taunt me with the contents of the said letter which no Lady would ever think of doing. She told me I should be the last one to complain about any thing, then I taxed her with reading my letter the answer the Matron made me she would be glad to get rid of me. I wished her to take me before the Committee, then she wished me to part friend. So I took her at her word, and left when my duty time expired at 8 p.m. My box went over the wall. Had the Curator been at his post he could have stopped me taking the train for London. But this is not the first box that disappeared over the Hospital wall perhaps will not be the last from the said Hospital. It seems a misterious place altogether when thoes in charge employ a Detective to watch a nurses proceedings. Thanking Matron for her great Interest in me, I am the Woman.

316, Cable Street, Shadwell.

### THE COST OF PROVISIONS.

"Another Matron" writes: I have read with deep interest and unqualified approval the letter on the cost of provisions by "A Provincial Matron" in The Hospital of October 13th. I was trained in one of the London hospitals, and filled a responsible position in a large provincial hospital, and have been matron in another, which had at first about 80, and latterly 150 beds. In the hospitals with which I was first connected nothing distressed me so much as the appalling waste which prevailed. What your correspondent did in her hospital of 21 beds I accomplished with 150, and there is no reason in the world why it should not be managed everywhere. We got out our working costs every month, the method of calculation being the same as that of your correspondent, and our average daily cost of provisions of all kinds, for patients, nurses, house servants, and staff, was generally under, and never exceeded, 10d. per head. But this can only be done by careful management and avoidance of waste. Although the scale of my rôle was much larger than that of your correspondent, there was really no difficulty. In efforts to secure economy the matron is naturally assisted or hindered by the sister in charge of the ward, and I have found myself unable to keep a sister, in every other respect well qualified, because she had no knowledge of domestic economy, and apparently no desire to acquire it. The question is of supreme importance, as most hospitals are, through the reduced annua value of their investments, becoming increasingly dependent on public support, and it is necessary that they should be able to show that their funds are wisely and judiciously administered. Hospital authorities should regard themselves as trustees administering public funds, and should watch their outgoings as scrupulously as if the money were their own. Those who are placed in positions of authority in hospitals should constantly remember that while efficiency must never be sacrificed to economy, it is a sacred duty to combine both. The thing is not only possible but easy.

### Dress and Uniforms.

By a Matron and Superintendent of Nurses.

Messrs. Denton and Holbrook, of Gloucester, are as usual well to the fore in their display of uniform for nurses. Especially tempting are the cloaks, for which this firm has earned a well-deserved reputation, and which we have no hesitation in considering to be one of the best for the money we have seen. It is perhaps difficult to pronounce an opinion as to whether the circular or the Russian cloak should bear the palm. Our own leaning has always been in favour of a well shaped circular, but since we have seen the plain Russian cloak made by Messrs. Denton and Holbrook we are rather inclined to waver, so particularly neat, nurse-like, and serviceable does it appear. A cloak of this description (see illustration) which they have just brought out in a thick

Outdoor Uniform from Messrs. Denton and Holbrook.

woollen cheviot at 25s. 9d. is delightfully warm and seasonable, and for the further sum of 2s. can be procured with sleeves in addition. The same shape in light summer serge may be had for 12s. 9d., or in a better quality at 19s. 9d. We cannot, however, pass by the "District" nurses' circular cloak, which is so excellent in shape and style that we most strongly recommend it to the notice of our readers. In light summer material it only costs 7s. 9d., and in a heavy soft winter cloth cannot be considered expensive at 16s. 11d. It should further be noted that the cloaks made by Messrs. Denton and Holbrook are thoroughly waterproof, and have in consequence an additional claim on the attention of those who have to be out in all weathers. Another specialité in which this firm deals is a particularly pretty "Princess" bonnet. It fits close to the head, the brim projecting just sufficiently to admit of a quilling of black velvet and a white border. This shape will be found becoming to most faces, and can be procured in all sizes simply trimmed with velvet for the sum of 4s. 6d.

### Boots and Shoes.

The London Shoe Company have recently issued a most alluring catalogue of their specialities for the coming season, and intending purchasers will not be disappointed as they look through its pages. We are glad to observe that the boots are all made with square, sensible, military heels, and appear to be as well adapted for comfort as they certainly are attractive to look upon. The price varies from 5s. 9d. to 23s. 9d. We are particularly fascinated by a delightful

Extra stout all blacking leather boot, leather lined, low heel and square toe, in three different fittings, price 18s. 9d.

Blacking leather golosh boot, to button or lace, in three shapes, toes pointed, medium, and square; also in half size, and three different fittings, price 12s. 9d. and 16s. 9d.

winter boot lined with soft white fur, and finished off round the top by a border of dark squirrel, the price of which is reasonable enough to place it within the reach of all. It would be hopeless with our limited space to attempt a description of the endless variety of shoes exhibited by this enterprising firm. Suffice it to say that they can be had in all sizes, shapes, prices, and qualities. They also combine ease with elegance in a remarkable degree. Fancy shoes are in bad taste when worn with uniform, but the plain glacé patent leather and kid are equally becoming, and are so well cut that they give a nice appearance to the foot, and are at the same time comfortable to the wearer. The premises of the London Shoe Company are so central (117, New Bond Street) that it will be within the power of most of our readers to pay them a visit *en passant*. The catalogue contains a table of sizes and fittings, which will be found very useful to customers at a distance.

### Notes and Queries.

### Queries.

(21) *Workhouse.*—Will you tell me through the medium of The Hospital, of which I am a constant reader, where to find particulars of workhouse infirmary nursing?—*Dorothy.*

(22) *Training.*—Advice on this subject is required by a girl of twenty-one.—*Mater.*

(23) *Salop.*—Is there an eye hospital in Shropshire?—*Inquirer.*

(24) *Ireland.*—Can you tell me where the Mercy Hospital and the Charitable Infirmary are to be found?—*Nurse Kate.*

(25) *Insane.*—Addresses wanted of lunatic asylums in Kent.—*Attendant.*

### Answers.

(21) *Workhouse (Dorothy).*—Write to the Hon. Secretary of Workhouse Infirmary Nursing Association, 6, Adam Street, Strand.

(22) *Training (Mater).*—We should advise the candidate to wait until she is twenty-four, and then enter her name at a good hospital or a workhouse infirmary which trains probationers.

(23) *Salop (Inquirer).*—Yes; at Shrewsbury.

(24) *Ireland (Nurse Kate).*—In Cork the latter is called the "North Charitable Infirmary and City of Cork General Hospital."

(25) *Insane (Attendant).*—You will find information in "Burdett's Hospital Annual."

### Wants and Workers.

Urgently wanted in a large district—old linen and calico; also a water-bed or large pillow for lending to bedridden patients. Nurse Catherine Kennedy, Wiemer Villa, Chase Side, Enfield.

For Nursing in India, Where to Go, and Minor Appointments, see overleaf.

### Nursing in India.

By Our Own Correspondent.

It is announced that Lady Elgin will accompany his Excellency the Viceroy on his tour through India this winter, visiting all the Zenana and Dufferin Hospitals en route, and this programme gives general satisfaction.

The Ripon Hospital, at Simla, especially arrested my attention during my travels this summer, for it certainly is one of the most picturesque buildings in the world. In spite of this its actual position is open to objection, for it stands within the native Bazaar, an arrangement designed presumably for the convenience of the sick who have the first claim on its charity. Like many of the houses in Simla the hospital is built on a Swiss model, and it is composed of three blocks, the centre one being devoted to the surgery, offices, and European and Eurasian patients.

The duties of the lady superintendent and nursing staff are confined to this block, and their quarters stand on one side of the hospital buildings. The native houses are in undesirable propinquity to the back, and they are inhabited by swarms of tightly-packed human beings.

The view from the front of the hospital and from the sisters' quarters is magnificent, embracing the snow-capped Himalayas, and some lower peaks which are also some thousands of feet high, and crowned by the hill stations of Jutoph, Subathu, and Kasauli, which are military depôts in the hot weather.

The nursing staff at the Ripon Hospital consists of the Lady Superintendent (a daughter of the late General Sir Herbert Macpherson), Sister Greenwood, and Sister Haywood (old Westminster pupils), and one or two other nurses. During the summer months they are all employed at the hospital, and paid by it, but during the winter they are free to do private nursing on their own account in the plains.

I believe while away from hospital duties in Simla they receive no pay or allowances from the Ripon Hospital, but they have the benefit of their own earnings.

In the European part of the building there are a few wards with six beds, and a number of private wards opening into a charming verandah that looks out at the hills. These rooms contain only one bed each, and the minimum charge is 5 Rs. per diem, ice, ærated waters, and stimulants being extra. A young officer who was in one spoke well of the hospital, and most gratefully of the nursing staff. Should the "Up-country Nursing Association" be started, I should suggest the Ripon Hospital as one of the headquarters for the nurses, the accommodation is so roomy, and there is space for at least four additional workers.

I should also recommend in connection with the Up-country Nursing Association that they should have associates attached to them, who, by paying an annual subscription to cover expenses, would be entitled to be sent for if a nurse was required. Nurses working on their own account would register their names at their nearest centres, say Mayo Hospital, Lahore; Ripon Hospital, Simla; or, for those south of the Punjaub, at Ramsay Hospital, Naini Tal. By the aid of the telegraph these nurses could be communicated with at once. A case arose this summer at Simla when a nurse was urgently required for an infectious case. Not one was available in Simla. They telegraphed to Naini Tal, the next nursing institution, which involved a delay of about two days. Two nurses happened to be waiting for employment in Umballa, the nearest railway station to Simla; but this was not known, so a needless expense was incurred and precious time wasted.

I heard of a nurse last winter going down to Lucknow, hoping to get work; but she did not make herself known to the right people, and a man was telegraphing unsuccessfully meanwhile for help for an urgent case. Yet the right person was within reach all the time.

At Yotoph there is a clean and orderly military hospital standing in a garden of roses on a hillside. A large leper asylum at Subathu is managed by the American Presbyterian Mission.

### Where to Go.

The New Gallery.

The fourth annual exhibition of the Society of Portrait Painters is now open to the public. Each year we notice its character becoming more internatinal, and certainly the present exhibition, is not confined by any insular limitation. For, though the portraits here exhibited are for the most part by English painters of the hour, yet these are supplemented by many specimens of work from foreign schools. For instance, the picture which has attracted most attention is of non-British origin. This Monsieur Gandara's "Princess de Chimay," which all Paris was talking about not long ago. The painter of this canvas is by birth a Spaniard, an artist with all the passion and spontaneousness of the south, which temperament has been supplemented by the severe discipline of the Paris schools. It would have been a venturesome thing, in other hands, to paint a life-size portrait in the manner in which this has been treated. With so few accessories, if we may use the term, and so simple a *mise en scène*. For the Princess stands there, quietly in her gleaming satin dress, unrelieved by any distracting *entourage*. One's eye rests on her and on her alone. Monsieur Gandara would have proved himself to be an artist of no low degree if his reputation depended alone on thi beautiful picture. The English art world will be charmed by it little less, it may fairly be assumed, than were the Parisian —and all Paris has been at its feet. Mr. Shannon is well represented in his portraits this winter; one or two of these are especially delightful. Professor Herkomer positively frightens us with his "Letty Lind," No. 130, in the South Room—a canvas—this is of vast proportions—on which is a preponderance of blue paint, lavishly administered, but which neither makes a portrait nor a picture. The fairy-like whirl of skirts with which we associate the original has failed entirely to be represented by Professor Herkomer. The portrait of his nephew is simpler, and a greater success in every way. Mr. W. Llewellyn's "Portrait of a Baby" is a beautiful specimen of the admirable work of this rising artist. The Home Rule portrait of Mr. Gladstone is one of the most striking pictures in the exhibition; this is hung on the walls of the West Room (No. 12). Enclosed in the frame is a copy of the Home Rule Bill itself, with Mr. Gladstone's autograph upon it. Other pictures in this exhibition also claim our interest and attention, which cannot be commented upon for want of space.

Trained Nurses' Club, 12, Buckingham-street, Strand.—Miss Wilson, President of the Midwives' Institute and Trained Nurses' Club, gives an "At Home" on Friday evening, 26th inst., to inaugurate the opening of the new club rooms.

### Minor Appointments.

Bona Convalescent Home.—Miss Catherine Keir, who has acted as Senior Charge Nurse in the Northern Infirmary, Inverness, for two years, has been unanimously elected Matron of the Infirmary's Convalescent Home at Bona. Miss Keir takes many good wishes to her new work from friends and fellow workers.

Guy's Hospital.—Miss Bessie Pitt Parret has been made Night Superintendent at this hospital, where she was trained, and afterwards worked as a staff nurse. Miss Parret has held an appointment in the Government Hospital at Poona, India, since May, and her return to her own hospital is a subject for congratulation to her old friends.

## Figures and Tables

**Figure f1:**
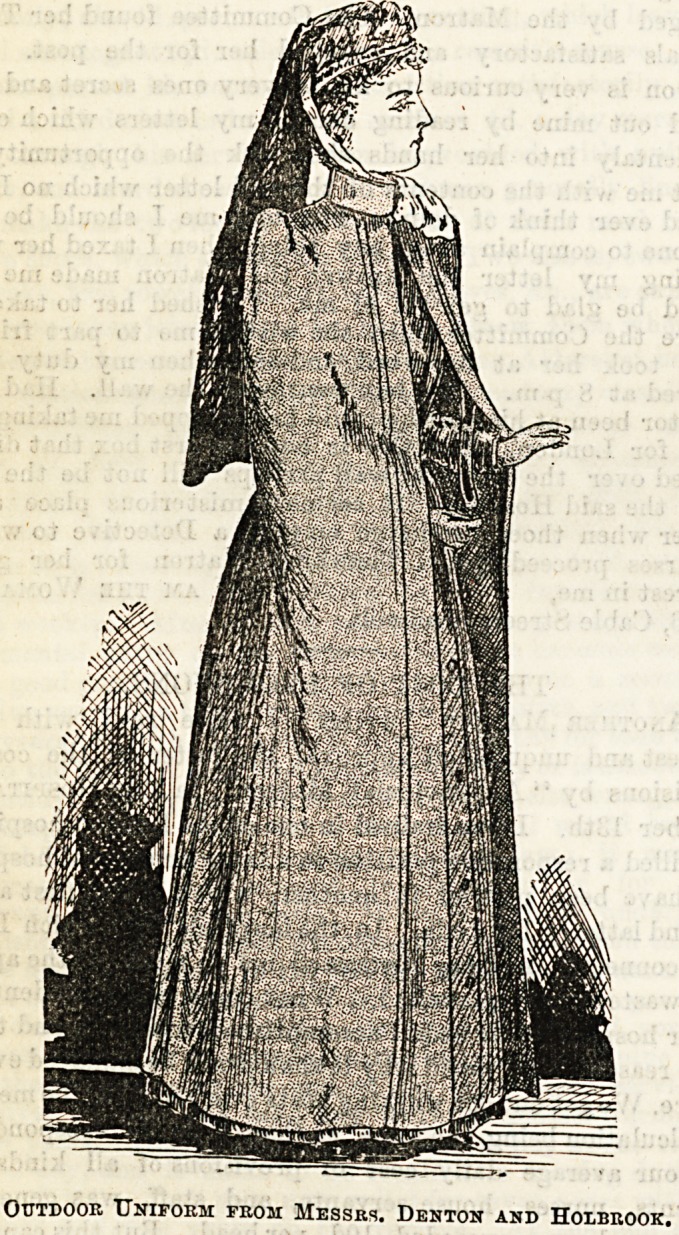


**Figure f2:**
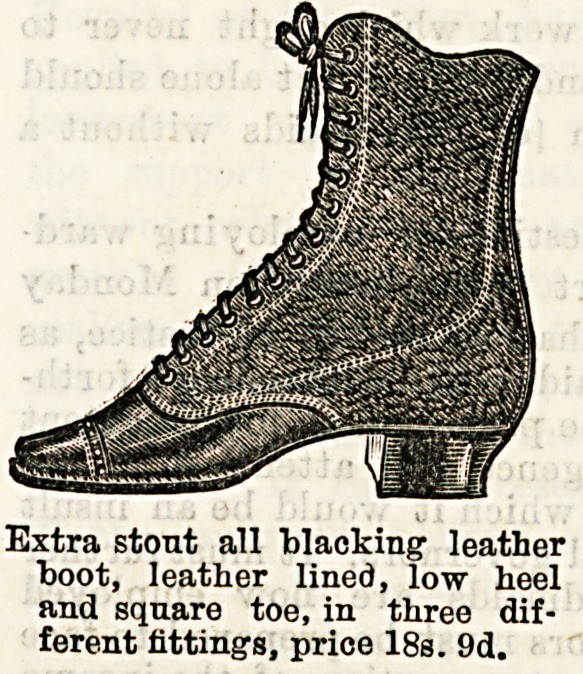


**Figure f3:**